# A mosaic of phenotypic variation in giant ragweed (*Ambrosia trifida*): Local‐ and continental‐scale patterns in a range‐expanding agricultural weed

**DOI:** 10.1111/eva.12614

**Published:** 2018-03-14

**Authors:** Stephen M. Hovick, Andrea McArdle, S. Kent Harrison, Emilie E. Regnier

**Affiliations:** ^1^ Department of Evolution, Ecology and Organismal Biology The Ohio State University Columbus OH USA; ^2^ Department of Horticulture and Crop Science The Ohio State University Columbus OH USA

**Keywords:** *Ambrosia trifida*, emergence timing, functional traits, intraspecific variation, local adaptation, seed size–seed number trade‐off, seed surface texture, seedling emergence

## Abstract

Spatial patterns of trait variation across a species' range have implications for population success and evolutionary change potential, particularly in range‐expanding and weedy species that encounter distinct selective pressures at large and small spatial scales simultaneously. We investigated intraspecific trait variation in a common garden experiment with giant ragweed (*Ambrosia trifida*), a highly variable agricultural weed with an expanding geographic range and broad ecological amplitude. Our study included paired populations from agricultural and natural riparian habitats in each of seven regions ranging east to west from the core of the species' distribution in central Ohio to southeastern Minnesota, which is nearer the current invasion front. We observed trait variation across both large‐ and small‐scale putative selective gradients. At large scales, giant ragweed populations from the westernmost locations were nearly four times more fecund and had a nearly 50% increase in reproductive allocation compared to populations from the core. The degree of surface texture on fruits also declined from east to west. Greater fecundity in the west represents a putative trade‐off between fruit size and fruit number across the study region, although no such trade‐off was found across individual plants. This pattern may effectively result in greater propagule pressure closer to the invasion front. At smaller spatial scales, plants from agricultural populations emerged later and were smaller than plants from riparian populations. However, because plants from agricultural populations allocated more biomass to reproduction, total fecundity did not differ across habitats. Our emergence data are consistent with previous observations showing delayed emergence in agricultural compared to natural populations; thus evolutionary change may be predictable as giant ragweed continues spreading into agricultural fields throughout North America. These shifts in life‐history strategy apparently bear no fecundity cost, suggesting that giant ragweed's success can be attributed at least in part to its substantial adaptive potential.

## 1 INTRODUCTION

Intraspecific trait variation is common in many species, reflecting processes such as local adaptation, phenotypic plasticity, and variable gene flow across the landscape (Albert, Grassein, Schurr, Vieilledent, & Violle, 2011). Particularly for species with large geographic distributions and ecological amplitudes, these processes may yield complex, non‐neutral spatial patterns of intraspecific variation (Bhattarai et al., 2017; Nelson & Anderson, 2015). A large geographic range enhances the breadth of bioclimatic variation a species encounters, and selection in response to such spatially continuous variation should result in trait autocorrelation among nearby populations (Murray, Brown, & Grace, 2003). On the other hand, large ecological amplitudes that allow a species to occupy distinct habitats may lead to local adaptation at small spatial scales (Hereford, 2009; Kittelson & Maron, 2001). Where distinct habitats occur repeatedly across a species' range, adaptation at large and small scales may occur simultaneously, yielding a pattern of continuous variation overlain by repeated occurrences of local adaptation. Whether and how a species partitions its phenotypic variation across such complex landscapes has implications for population establishment and persistence, range expansions, and evolutionary potential across the range (Forsman, 2014); however, such co‐occurring scale‐dependent patterns of variation are rarely documented (but see Délye et al., 2010).

Weedy and invasive species should be particularly useful for investigating patterns of morphological variation and adaptation at multiple spatial scales. Because many such species are geographically widespread and occur in a range of distinct habitats (e.g., Nelson & Anderson, 2015), they should experience a complicated mosaic of selection pressures. Many weedy and invasive species also harbor substantial genetic and/or phenotypic variability at the population level (Clements et al., 2004; Dlugosch & Parker, 2008; Lavergne & Molofsky, 2007; Vigueira, Olsen, & Caicedo, 2013; Warwick, Thompson, & Black, 1987), making it possible that spatial patterns of selection could yield corresponding patterns of phenotypic variation. Further, spatial patterns in phenotypic variation may repeat across the landscape, especially when species exist in commonly occurring environments with unique selection pressures such as agricultural fields, roadsides, and other highly disturbed areas (Lee & Gelembiuk, 2008; Vigueira et al., 2013). And, because range expansions and postintroduction population dynamics are inherently variable, populations from different parts of the weedy or invasive range may differ in their responses to selective pressures or the strength of selection.

Of course, despite the many reasons one might expect weedy and invasive species to exhibit complex patterns of phenotypic variation across their range, much of that variation could simply be due to phenotypic plasticity and not maintained when individuals are grown in a common environment. The ability to express traits plastically depending on local conditions is often seen as a key attribute of such opportunistic species (Baker, 1965; Davidson, Jennions, & Nicotra, 2011; Pyšek & Richardson, 2008; Richards, Bossdorf, Muth, Gurevitch, & Pigliucci, 2006). If phenotypic plasticity is the primary process underlying observed phenotypic variation, local adaptation will be much less likely because plasticity can effectively buffer populations against local selective pressures (De Jong, 2005). In this case, similar genotypes may achieve highly divergent phenotypes under variable abiotic or biotic conditions (Parker, Rodriguez, & Loik, 2003).

Although substantial research effort has gone into identifying which traits should be most important for success among weedy and invasive species (Pyšek & Richardson, 2008; Van Kleunen, Weber, & Fischer, 2010), much less emphasis has been given to within‐species variation (Albert et al., 2011), particularly across clinal gradients, distinct habitat types, or establishment centers versus invasion fronts. As a result, we may underestimate the importance of trait plasticity and/or rapid evolutionary change in the colonization success of widespread weedy or invasive species (Whitney & Gabler, 2008; Williams, Kendall, & Levine, 2016). Assuming single trait values to be static representations of a species with the ability to colonize a wide range of site conditions also leads to an under‐appreciation of intraspecific trade‐offs among traits. For example, fecundity is a commonly cited predictor of invasiveness in plants. But, because many species exhibit a seed size–seed number trade‐off (Fenner & Thompson, 2005), the use of a single trait value to characterize fecundity might overlook meaningful biology relevant to a species' ability to establish new populations or persist once established. And because large seeds are often better able to sustain developing seedlings in the presence of competing vegetation than are small ones (Leishman, Wright, Moles, & Westoby, 2000), it may be adaptive for plants growing in dense vegetation to produce large seeds—even if this means producing fewer of them. The general lack of knowledge about intraspecific trait variation in weedy and invasive species thus represents a major gap in understanding the ecology of these groups and a potential “Achilles heel” in our ability to predict their adaptive responses to changing conditions.

The weedy species giant ragweed (*Ambrosia trifida*, Asteraceae) provides a compelling system for investigating morphological variation across multiple spatial scales. Giant ragweed is a wind‐pollinated and mostly outcrossing species that is native to North America (Bassett & Crompton, 1982) and can be found throughout much of the continent (Payne, 1970). The species is highly variable morphologically and genetically (Abul‐Fatih, Bazzaz, & Hunt, 1979; Patzoldt & Tranel, 2002; Sako et al., 2001); thus, we expect it should respond to variable selection pressures across the range. In its native habitats, giant ragweed generally occurs in early‐successional and disturbed sites with moist soils (Bassett & Crompton, 1982), although it also occurs in drier upland sites (Regnier et al., 2016). Giant ragweed is also a problematic agricultural weed that causes substantial crop losses when not controlled early in the season (Barnett & Steckel, 2013; Ganie et al., 2017; Harrison, Regnier, Schmoll, & Webb, 2001; Webster, Loux, Regnier, & Harrison, 1994) and that has been found to have multiple instances of herbicide resistance (Heap, 2018). It has been a management concern for farmers in the Eastern Corn Belt for at least the past 30 years, but more recently it has been undergoing range expansion in both agricultural and successional habitats farther west and north into the Great Plains where the climate is drier and cooler and where historical agricultural practices differ (Regnier et al., 2016). Whether its recent range expansion has resulted from natural versus anthropogenic dispersal is not known, leaving important questions unanswered regarding the source of newly weedy giant ragweed in crop fields. Selective pressures almost certainly vary across giant ragweed's range, at both large and small scales.

Seedling emergence patterns in giant ragweed provide valuable initial clues regarding habitat‐specific responses to selection at small scales, while also accounting for larger‐scale patterns of variation throughout its expanding range. Common garden experiments have shown that seedling emergence in ragweed populations from successional habitats is early and brief but in agricultural habitats is more prolonged (Davis et al., 2013; Hartnett, Hartnett, & Bazzaz, 1987; Schutte, Regnier, & Harrison, 2012), indicating strong selection for early, coordinated emergence in heavily vegetated sites that becomes relaxed when ragweed invades nearby cultivated fields. These studies were relatively limited in spatial scale (single sites, or up to a ~500 km^2^ region in west‐central Ohio) and centered on the core of giant ragweed's range; thus, it is unknown whether such differences in emergence phenology also occur farther west where giant ragweed is more recently established as a problematic weed (Regnier et al., 2016). If habitat‐specific emergence patterns are not widespread, this would suggest local adaptation has not yet occurred, perhaps because the necessary phenotypic variation has not been introduced to these regions or because not enough time has passed for populations to become locally adapted.

For this study, we used a common garden experiment to test the hypothesis that giant ragweed populations differ in key traits at both large and small spatial scales. We quantify trait variation across the species' range and among populations from two common but distinct habitat types: agricultural fields and early‐successional riparian habitats. We focus on multiple traits that are common indicators of success in weedy and invasive species, in addition to precisely quantifying fruit morphology including the degree of surface texture. Although adaptive benefits of surface texture in plant dispersules are largely speculative, giant ragweed is notably variable for these traits (Sako et al., 2001). For all of these traits, we test whether giant ragweed exhibits (i) large‐scale patterns of variation associated with broad geographic gradients and (ii) small‐scale patterns of variation associated with differences in habitat.

## 2 METHODS

### 2.1 Seed collections

**Figure 1:**
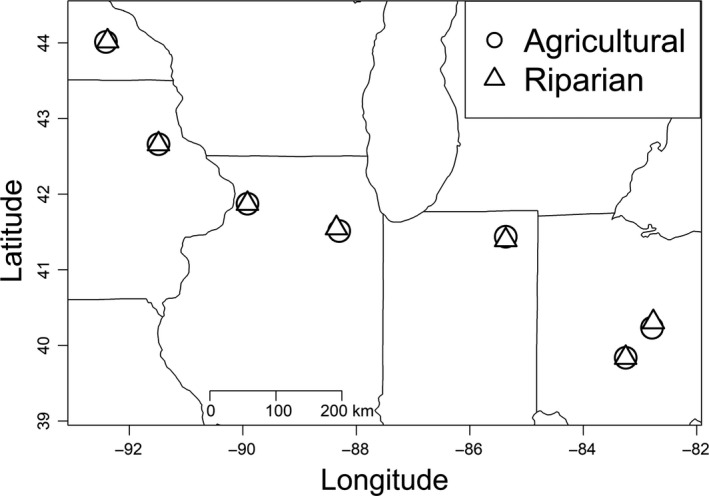
Map showing locations of ragweed source populations used in the present study. Paired agricultural and riparian populations were selected from each of seven regions (*n *=* *14 populations total)

Fruits (involucres, each enveloping a single achene) were collected from paired riparian and agricultural populations in each of seven regions spanning from Ohio to southeastern Minnesota and northeastern Iowa (*n *=* *14 populations) in the fall of 2011. Populations within region were separated by at most 8.5 km (average: 3.2 km), and our closest regions were separated by 60 km (Figure 1). Fruits were collected separately for 25 plants per population and kept at room temperature until extraneous plant material was removed (~12 weeks), after which they were stored at 4°C.

### 2.2 Common garden experiment

To reduce maternal effects, plants were grown under common conditions in a greenhouse at Ohio State University during the summer of 2014. Fruits were stratified for 70 days in moist sand at 4°C, planted into plug trays on June 25 and 26 and then transplanted into 18.9‐L pots once they had initiated at least two true leaves. Plants were fertilized weekly in plug trays and twice weekly in the larger pots using 200 ppm of 20‐10‐20 fertilizer. For each maternal family, we recorded time to emergence (in days) of the first seedling out of three planted fruits and retained that plant for all subsequent measurements.

Giant ragweed is wind‐pollinated and primarily outcrossing, so to keep pollen movement restricted within populations once flowering began, we enclosed plants from the same population in tents made of Tyvek® HomeWrap® (DuPont, Wilmington, DE, USA) hung from a PVC frame. Tyvek is vapor‐permeable but with a pore size small enough to limit pollen movement (Gitz, Baker, Xin, Burke, & Lascano, 2015; Smith & Mehienbacher, 1994). Enclosures were shaken occasionally from the outside to facilitate pollen movement among individuals from the same population. Once plants from a given population began shedding ripe fruits, we recorded final plant height and then collected fruits and aboveground biomass. Populations were harvested in the same order they had been tented. All aboveground biomass was dried at 60°C and weighed, except for fruits, which were weighed fresh and counted to estimate fecundity. Total aboveground biomass for each plant was estimated as the sum of total fruit biomass plus dried nonfruit biomass. Reproductive allocation was calculated as the ratio of total fruit biomass to total aboveground biomass.

### 2.3 Fruit morphology metrics

**Figure 2:**
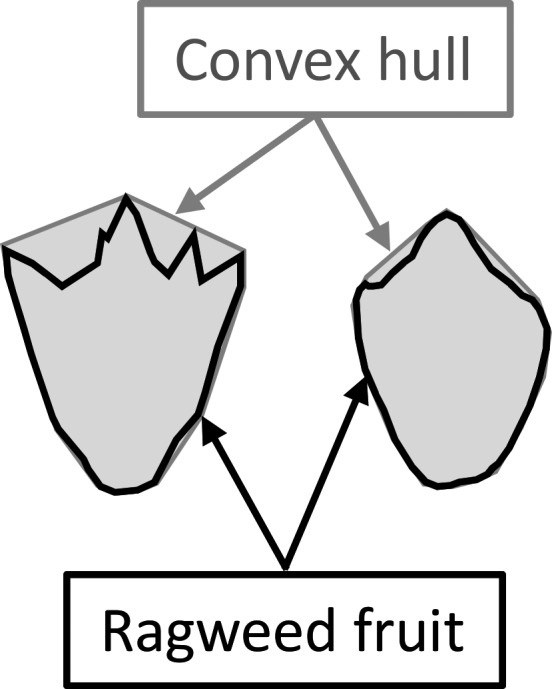
Ragweed fruit surface texture is highly variable, as illustrated here with two representative samples. Surface texture was quantified using two indices, each of which is based on the outline of an individual fruit and the convex hull subsuming that outline. Convexity is calculated as convex hull perimeter/fruit perimeter, and solidity is calculated as the fruit area/convex hull area; both indices are bounded by 0 and 1, with large values indicating relatively little surface texture. In the examples shown here, the fruit on the left is more textured (solidity = 0.813 and convexity = 0.791) than the fruit on the right (solidity = 0.932 and convexity = 0.949)

Both generations of fruits (field‐collected and greenhouse‐grown) were scanned and measured to assess variation in fruit morphology. We randomly selected five undamaged fruits per maternal plant for 25 maternal plants per population from the field‐collected fruits and up to 18 maternal plants per population from the greenhouse‐grown fruits (range: 7–18, due to poor germination and/or fruit production by some families). We scanned fruits with an Epson 10000 Excel scanner and processed images of individual fruits using ImageJ 1.48V (Rasband, 1997) to quantify fruit area, length:width ratios, and two indices of fruit surface texture: convexity (convex hull perimeter divided by fruit perimeter) and solidity (fruit area divided by convex hull area). Both indices have values bounded by 0 and 1, with large values indicating relatively little surface texture (Olson, 2011; see Figure 2). We also estimated individual fruit mass, weighing sets of five fruits from each maternal plant together and averaging.

### 2.4 Viability assessment

For greenhouse‐grown fruits only (which were somewhat more variable morphologically than field‐collected fruits), we assessed viability using X‐ray imagery (Del'Aquila, 2007) at the Ornamental Plant Germplasm Center at Ohio State University to determine whether morphology was influenced by the degree of embryo development and to determine whether viability varied based on region or habitat of origin. Fruits were manually scored for viability based on whether the embryo filled at least 75% of the seed case.

### 2.5 Statistical analysis

All analyses were performed in R version 3.2.3 (R Core Team 2016), unless noted below. Statistical models were constructed by first fitting a fully defined model with all predictors of interest, then sequentially using log‐likelihood tests to justify dropping individual predictors. We imposed two restrictions on model simplification: first, that lower‐order interactions could be dropped only if they were not a component of higher‐order interactions, and second, that our main effects of interest were always retained in the model. Inferences are based on Type III sums of squares from the *car* package. In all cases, residuals were approximately normally distributed.

From the common garden experiment, our primary goals were to test the main effects of habitat (agricultural versus riparian), region (using longitude of the source population as a continuous indicator of location), and their interaction. We analyzed data on time to emergence, total aboveground biomass, maximum height, proportional biomass allocation to reproduction, and total fecundity. Time to emergence, aboveground biomass, and fecundity data were log_10_‐transformed.

From the fruit size and morphology dataset, our goals were the same as those from the common garden experiment (assessing the effects of habitat, region, and their interaction), in addition to assessing year effects (fruits from the field‐collected versus greenhouse‐grown generation) plus year interactions with habitat and longitude. We analyzed fruit morphology data using nontransformed family‐level means for four key responses: fruit mass, solidity, convexity, and length:width ratios. Preliminary analyses indicated that fruit area was correlated with solidity, convexity, and length:width ratios, so for all analyses on those responses we included fruit area as a covariate. Individual fruit mass and area were highly correlated (*r *=* *.73, *p *<* *.001), so we present results from only the former response. We also analyzed the proportion viable fruits (based on X‐rayed images), but as these data were collected from greenhouse‐grown plants only (see above), the effect of year and interactions with year were not tested.

We used PCA to facilitate the interpretation of changes in fruit morphology across space and time. Our input variables were family‐level mean fruit mass, fruit area, convexity, solidity, and length:width ratios (all centered and scaled).

We used variance partitioning (proc MIXED in SAS version 9.2; SAS Institute 2008) to assess the degree to which variability in fruit size, and morphology was partitioned by region, habitat (nested within region), and maternal family (nested within population). We assessed only responses where data were available from individual fruits (rather than family‐level means) because a key goal of these analyses was to differentiate between variation within versus among maternal families. For these analyses, region was treated as a categorical variable and data from field‐collected and greenhouse‐grown fruits were analyzed separately.

We estimated narrow‐sense heritability in fruit size and morphology traits with parent–offspring regressions, basing inferences on bootstrapped 95% confidence intervals. Mid‐parent trait values were not available (pollen donors were not controlled); thus, these values likely overestimate true heritabilities.

## 3 RESULTS

### 3.1 Traits of greenhouse‐grown plants

**Figure 3:**
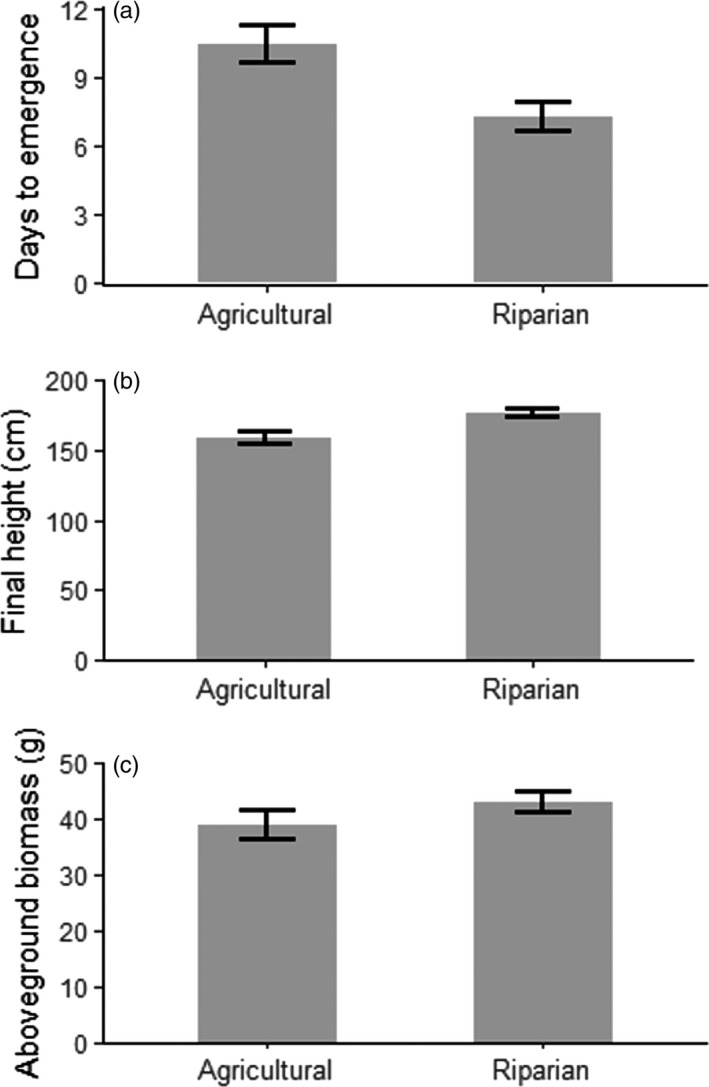
Trait variation in greenhouse‐grown giant ragweed, based on source habitat. All habitat‐based differences are statistically significant (Table 1). Data are averaged across regions. Error bars are ±1*SEM*

**Figure 4:**
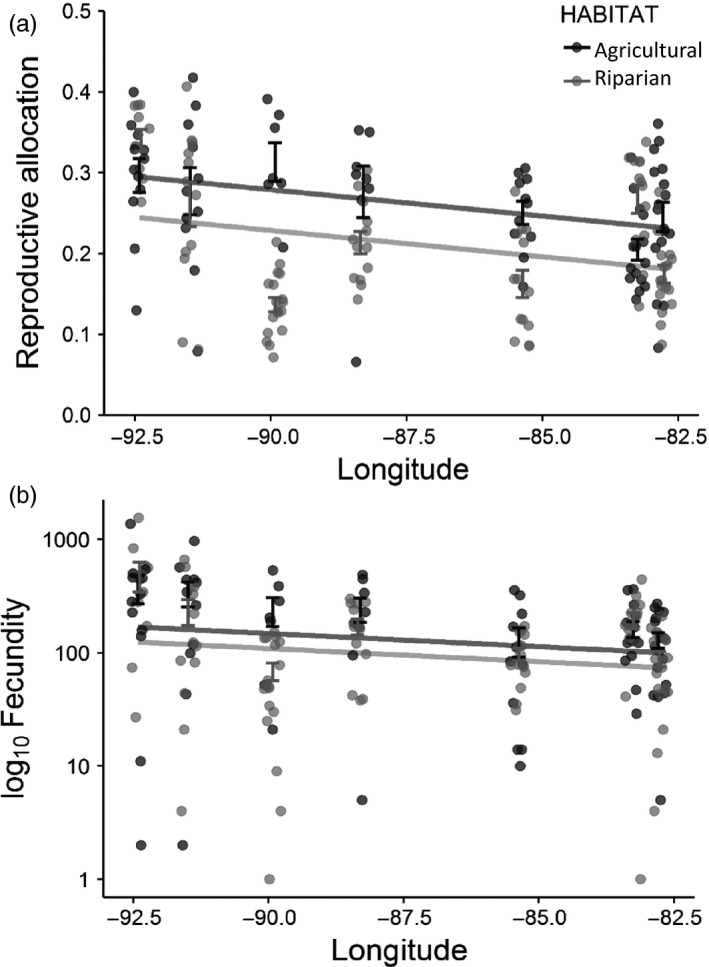
Variation in reproductive allocation and fecundity in greenhouse‐grown plants, based on source habitat and region. Population means ± 1*SEM* are shown. Points represent data from all individuals in the experiment, jittered slightly to facilitate interpretation. Lines represent model‐based parameter estimates as reported in Table 1

Despite the close proximity of our paired riparian and agricultural populations, greenhouse‐grown plants differed in a number of ways based on source habitat. The first seedlings from riparian populations emerged in 7.3 days on average compared to 10.4 days for seedlings from agricultural populations (Table 1; Figure 3a; see Figure S1 for population means). The resulting plants from riparian populations were 10.0% taller and produced 9.4% more total biomass than plants from agricultural populations (Figure 3b,c), but because plants from agricultural habitats allocated proportionally more biomass to reproduction (Figure 4a), total fecundity did not differ by habitat type (Figure 4b; Table 1; see Figure S1 for population means).

**Table 1:** Results from reduced models used to analyze data collected from greenhouse‐grown plants. Positive parameter estimates for the habitat effect indicate an increase in populations from riparian relative to agricultural habitats, and positive parameter estimates for the longitude effect indicate an increase in eastern relative to western populations

	Parameter estimate (SE)	SS	*df*	*F*	*p*
Time to emergence (log_10_ days)					
Model (*R* ^2^_adj_ = .043)	–	–	2,177	5.02	.008
Intercept	1.21 (0.64)	0.47	1,177	3.55	.061
** Habitat**	**−0.17 (0.055)**	**1.27**	**1,177**	**9.59**	**.002**
Longitude	0.004 (0.007)	0.04	1,177	0.26	.608
Final height (cm)					
Model (*R* ^2^_adj_ = .054)	–	–	2,177	6.09	.003
** Intercept**	**216.88 (61.88)**	**15149**	**1,177**	**12.28**	**<.001**
** Habitat**	**17.93 (5.27)**	**14285**	**1,177**	**11.58**	**<.001**
Longitude	0.665 (0.710)	1081	1,177	0.88	.350
Aboveground biomass (log_10_ g)					
Model (*R* ^2^_adj_ = .029)	–	–	2,177	3.66	.028
** Intercept**	**1.65 (0.55)**	**0.87**	**1,177**	**9.08**	**.003**
** Habitat**	**0.13 (0.047)**	**0.70**	**1,177**	**7.28**	**.008**
Longitude	0.002 (0.006)	0.01	1,177	0.11	.737
Reproductive allocation (proportion)					
Model (*R* ^2^_adj_ = .150)	–	–	2,177	16.79	<.001
** Intercept**	−**0.31 (0.14)**	**0.03**	**1,177**	**4.96**	**.027**
** Habitat**	−**0.05 (0.01)**	**0.11**	**1,177**	**18.31**	**<.001**
** Longitude**	−**0.007 (0.002)**	**0.10**	**1,177**	**16.88**	**<.001**
Fecundity (log_10_ fruits)					
Model (*R* ^2^_adj_ = .025)	–	–	2,177	3.28	.040
Intercept	0.07 (0.98)	0.002	1,177	0.01	.943
Habitat	−0.13 (0.08)	0.78	1,177	2.53	.113
** Longitude**	−**0.023 (0.011)**	**1.33**	**1,177**	**4.32**	**.039**

SE, standard error; SS, sums of squares; *df*, degrees of freedom; Significant effects (*p *<* *.05) are highlighted in bold.

Plants from the westernmost populations were nearly four times more fecund and had a nearly 50% increase in reproductive allocation compared to eastern populations (Figure 4), regardless of source habitat (see Table 1). Based on X‐ray images of ragweed fruits, there were no differences in apparent viability based on either habitat or longitude (all *p *>* *.13; data not shown).

### 3.2 Fruit mass and morphology

**Figure 5:**
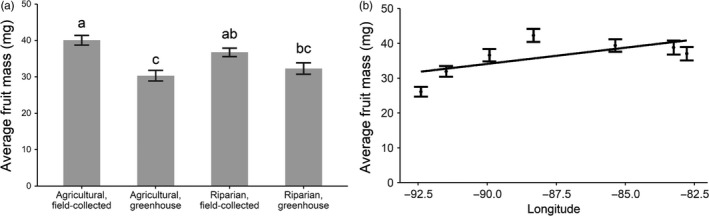
Ragweed individual fruit mass varied (a) by year and source habitat and (b) by source region, with smaller fruits in the west relative to the east of our sample range. Pairwise differences in (a) are based on Tukey comparisons with α = 0.05. The best‐fit line in (b) represents model‐based parameter estimates as reported in Table 2. Error bars are ±1*SEM*

On average, individual fruit mass was greater from agricultural compared to riparian populations and from 2014 field collections compared to 2015 greenhouse‐grown plants, although the magnitude of the year effect was more pronounced for agricultural than for riparian populations (significant Habitat × Year interaction; Table 2 and Figure 5a). Individual fruit mass also varied across our sample range; fruits from the westernmost population were 42% smaller than those from the easternmost population, resulting in an average decrease in fruit mass of 0.93 mg per degree of longitude (Table 2, Figure 5b). Taken together with the longitudinal variation in fecundity we observed (see above), this pattern of variation in fruit mass suggests a potential trade‐off between fruit number and size. However, individual fruit mass in greenhouse‐grown plants was not correlated with fecundity at the individual plant level (all *p *>* *.1, including analyses on the full dataset as well as subsets by source habitat, region, and population).

**Table 2:** Results from reduced models used to analyze data collected from field‐collected and greenhouse‐grown fruits

	Parameter estimate (SE)	SS	*df*	*F*	*p*
Individual fruit mass (mg)					
Model (*R* ^2^_adj_ = .088)	–	–	4,525	13.7	<.001
** Intercept**	**121.9 (16.2)**	**0.013**	**1,525**	**56.48**	**<.001**
** Habitat**	**−3.4 (1.6)**	**0.001**	**1,525**	**4.14**	**.043**
** Longitude**	**0.93 (0.19)**	**0.006**	**1,525**	**25.57**	**<.001**
** Year**	**−10.4 (2.1)**	**0.006**	**1,525**	**24.98**	**<.001**
** Habitat × Year**	**5.6 (2.8)**	**0.001**	**1,525**	**3.95**	**.047**
Solidity					
Model (*R* ^2^_adj_ = .117)	–	–	5,524	18.44	<.001
** Intercept**	**0.864 (0.035)**	**0.589**	**1,524**	**610.37**	**<.001**
Habitat	0.002 (0.003)	0.001	1,524	0.4	.525
Longitude	−0.0005 (0.0004)	0.001	1,524	1.54	.216
** Year**	−**0.022 (0.003)**	**0.054**	**1,524**	**56.5**	**<.001**
** Fruit area**	−**0.001 (0.0002)**	**0.015**	**1,524**	**15.66**	**<.001**
Convexity					
Model (*R* ^2^_adj_ = .146)	–	–	4,525	23.67	<.001
** Intercept**	**0.818 (0.046)**	**0.52727**	**1,525**	**318.59**	**<.001**
Habitat	−0.001 (0.004)	0.00025	1,525	0.15	.698
** Longitude**	−**0.001 (0)**	**0.01396**	**1,525**	**8.44**	**.004**
** Year**	−**0.018 (0.004)**	**0.03981**	**1,525**	**24.06**	**<.001**
** Fruit area**	−**0.002 (0)**	**0.08791**	**1,525**	**53.12**	**<.001**
Length:Width ratio					
Model (R^2^_adj_ = .098)	–	–	4,525	15.41	<.001
** Intercept**	**2.207 (0.242)**	**3.84**	**1,525**	**83.16**	**<.001**
Habitat	0.018 (0.019)	0.04	1,525	0.88	.349
Longitude	0.003 (0.003)	0.05	1,525	1.17	.280
** Year**	−**0.123 (0.02)**	**1.78**	**1,525**	**38.59**	**<.001**
** Fruit area**	−**0.007 (0.001)**	**1.31**	**1,525**	**28.4**	**<.001**

Positive parameter estimates for the habitat effect indicate an increase in populations from riparian relative to agricultural habitats, positive parameter estimates for the Longitude effect indicate an increase in eastern relative to western populations, and positive parameter estimates for the year effect indicate an increase in the greenhouse‐grown relative to the field‐collected generation. SE, standard error; SS, sums of squares; *df*, degrees of freedom. Significant effects (*p* < .05) are highlighted in bold.

**Figure 6:**
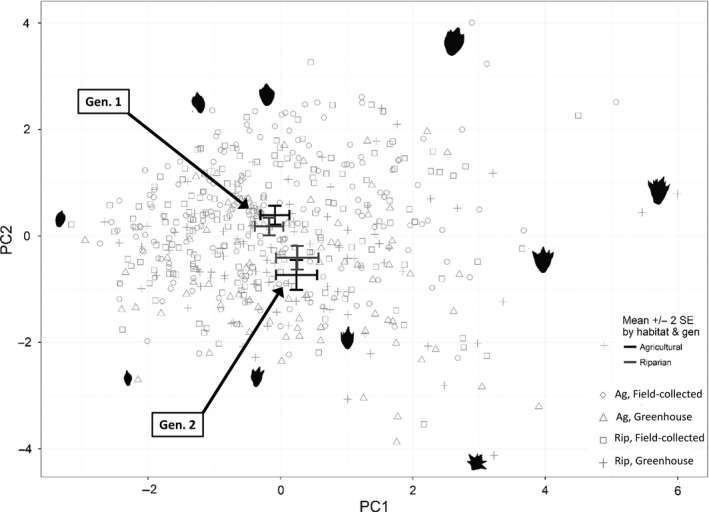
Principal components analysis of individual fruit size, morphology, and surface texture values, including fruits from both the field‐collected (Gen. 1) and greenhouse‐grown generations (Gen. 2). Error bars depict ±2*SE* around mean PC1 and PC2 scores by source habitat and year. Images of individual fruits associated with specific data points are provided to illustrate the main axes of variation. All fruit images are to scale. Table S2 for PC loading values

Fruit morphology varied by year and, to a lesser extent, by longitude and habitat. Solidity and convexity values were lower in fruits collected from greenhouse‐grown plants compared to the field‐collected maternal parents, reflecting greater surface texture in the offspring generation (Table 2). Convexity values (but not solidity) were greater in plants from the western relative to eastern populations (Figure S2; Table 2), reflecting somewhat reduced surface texture in the west. Length:width ratios indicated that ragweed fruits were 6.4% more elongated in the field‐collected generation relative to the greenhouse‐grown generation (Figure S3; see also Figure 6). Fruit area was correlated with all metrics of fruit surface texture and morphology (Tables 2 and S1), such that larger fruits were both more textured and less elongated (Figure 6).

Nearly 73% of the variation in fruit size and morphology was described by the first two axes of PCA (Table S2). The first PC axis reflected the positive correlation between individual fruit size (area and mass) and fruit surface texture (low convexity and solidity values; see Figure 6 and PC1 loadings in Table S2). The second PC axis was also related to fruit size and surface texture, but reflected another dimension of variability in which fruit surface texture and fruit size were negatively correlated for a subset of fruits (Figure 6; Table S2). This corresponds with our finding from univariate analyses that fruits were smaller on average yet still more textured in the greenhouse‐grown relative to the field‐grown generation.

### 3.3 Heritability and variance partitioning of fruit size and morphology traits

The majority of variation in fruit size and morphology was partitioned within maternal families (Table 3), regardless of whether maternal plants had been grown in the same environment or not. From greenhouse‐grown plants, a significant amount of variability in fruit surface texture was also explained by source habitat.

**Table 3:** Results from variance partitioning analysis on fruit size and morphology data, where variance was partitioned by study region, source habitat (nested within region), and maternal family (nested within the region × habitat combination)

	Field‐collected		Greenhouse‐grown	
	Percent total variance	*p*	Percent total variance	*P*
Fruit area				
Region	9.63	.093	7.59	.114
Habitat(Region)	**7.46**	**<.001**	3.61	.125
Family(Region×Habitat)	**57.74**	**<.001**	**63.4**	**<.001**
Residual	25.17		25.4	
Solidity				
Region	**5.86**	**.001**	−7.39	.902
Habitat(Region)	−1.99	.96	**16.31**	**.001**
Family(Region × Habitat)	**68.09**	**<.001**	**62.06**	**<.001**
Residual	28.03		29.02	
Convexity				
Region	**8.37**	**.003**	−0.77	.538
Habitat(Region)	−1.37	.775	**10.2**	**.007**
Family(Region × Habitat)	**71.01**	**<.001**	**59.34**	**<.001**
Residual	21.99		31.23	
Length:Width ratio				
Region	0.89	.393	−2.31	.843
Habitat(Region)	**5.16**	**.005**	2.87	.151
Family(Region × Habitat)	**57.75**	**<.001**	**53.84**	**<.001**
Residual	36.2		45.6	

Analyses were conducted separately on field‐collected and greenhouse‐grown fruits. Significant effects (*p *<* *.05) are highlighted in bold.

Estimates of narrow‐sense heritability were overall quite high (from 0.44 to 0.98, excluding one estimate that did not differ from zero) and did not vary by habitat type (see Table 4). Heritability for individual fruit mass was lower than for our other morphology metrics, but because of the wide confidence intervals these values did not differ. All heritability estimates were significantly greater than zero except for individual fruit mass in agricultural populations (Table 4).

**Table 4:** Narrow‐sense heritability estimates and bootstrapped 95% confidence intervals (CI). All estimates are significantly greater than zero except for individual fruit mass in agricultural populations

	All populations (*n *=* *169)		Agricultural populations (*n *=* *78)		Riparian populations (*n *=* *91)	
	*h* ^2^	95% CI	*h* ^2^	95% CI	*h* ^2^	95% CI
Individ. fruit mass	0.44	(0.132, 0.740)	0.27	(−0.186, 0.726)	0.60	(0.192, 1.000)
Fruit area	0.96	(0.688, 1.232)	0.86	(0.444, 1.276)	1.04	(0.678, 1.406)
Convexity	0.90	(0.624, 1.176)	0.84	(0.420, 1.252)	0.94	(0.564, 1.316)
Solidity	0.91	(0.632, 1.184)	0.85	(0.438, 1.270)	0.98	(0.612, 1.348)
Length:Width ratio	0.75	(0.462, 1.038)	0.75	(0.328, 1.176)	0.77	(0.378, 1.162)

## 4 DISCUSSION

Based on plants grown in a common environment, we observed striking large‐scale variation in fruit size and putative life‐history trade‐offs related to reproduction in giant ragweed: plants from the west allocated more biomass to reproduction and were four times more fecund than those from the east, producing individual fruits that were smaller and had somewhat less surface texture. We also observed recurrent differences in phenology, plant size, and biomass allocation based on local land‐use patterns: seedlings from riparian populations emerged sooner and the resulting plants were larger in comparison with plants from agricultural populations. The variability we observed at both spatial scales appears to have a genetic basis rather than being solely a response to environmental variation. This is particularly likely for fruit size and morphology, as we reduced maternal effects in those traits by crossing plants in the greenhouse and collecting data from the resulting fruits. Below, we present testable hypotheses regarding potential causes for these differences and discuss implications of the mosaic patterns in phenotype we identified in this economically important and range‐expanding weedy species.

### 4.1 Regional‐scale variation in fruit size and number

To our knowledge, ours is the only system in which geographic variation in both fruit size and number has been documented across a substantial portion of a species' range. We note that at smaller scales a similar pattern has been reported in *Prunus virginiana* growing in distinct habitats. In Montana, USA, *P. virginiana* individuals in riparian habitats produce a greater number of smaller seeds compared to those in moisture‐limited slope habitats, presumably reflecting variable seedling recruitment in low‐ versus high‐stress environments (Parciak, 2002). Although patterns in both giant ragweed and *P. virginiana* imply a trade‐off between fruit number and fruit size, correlations between these responses at the individual level (either within or among populations) were not consistently negative, as might be predicted from theory (Smith & Fretwell, 1974). Despite the commonness of seed size–seed number trade‐offs both among species (Jakobsson & Eriksson, 2000; Turnbull, Rees, & Crawley, 1999) and within species (Ågren, 1989; Eriksson, 1999; Lázaro & Traveset, 2009), the lack of such a trade‐off within species is also common (Michaels et al., 1988; Mojonnier, 1998; Willis & Hulme, 2004; Sõber & Ramula, 2013; see also review by Moles, Falster, Leishman, & Westoby, 2004). The absence of an intraspecific seed size–number trade‐off for many species may reflect a greater degree of plasticity in seed number than seed size in response to variable resource availability (Harper, Lovell, & Moore, 1970; Paul‐Victor & Turnbull, 2009). Therefore, rather than treating regional variation in fruit size and number as a single syndrome we mostly consider both components separately in the following text, focusing first on fruit size.

The geographic variation in giant ragweed fruit size we observed has been documented previously, based on field collections from throughout North America (Payne & Jones, 1962). However, no common garden experiments were performed at the time to determine whether those patterns had a genetic basis. Unlike the *P. virginiana* system described above (Parciak, 2002), the extent to which fruit size variation may be adaptive across the range of giant ragweed is unclear. We present two potential mechanisms by which variable selective pressures across our study region could have led to these large‐scale patterns in fruit size.

First, larger fruits in the east may be adaptive because of biotic selective pressures related to postdispersal seed predation and/or secondary dispersal. Seed predator exclusion experiments in Ohio have shown predation‐related losses of giant ragweed fruits reaching nearly 90% (Harrison, Regnier, & Schmoll, 2003), indicating the potential for strong effects on local population dynamics and phenotypic evolution. In that study, smaller fruits were preferred by invertebrates and larger fruits by small rodents, but it is not known whether variable seed predation by these two key groups has influenced fruit size across giant ragweed's range. Giant ragweed fruits are also collected by the non‐native earthworm *Lumbricus terrestris*, facilitating enhanced recruitment from (thus selection for) larger fruits because smaller fruits are buried more deeply in the soil than is optimal for their emergence (Regnier et al., 2008). This association is common in the eastern part of our study region (Schutte, Liu, Davis, Harrison, & Regnier, 2010) and could contribute to selection for larger fruits there. We do not know how common these interactions between ragweed and earthworms (or other key seed predators/dispersers) are throughout its range, but such data would be useful for testing this hypothesis.

Second, variation in fruit size across our region could reflect abiotic selective pressures due to climate. For example, smaller fruits may be adaptive in the colder and drier conditions farther north and west in our study region if they are less prone to desiccation or require less water for imbibition to trigger germination relative to larger fruits. Seed size‐dependent patterns of seedling survival may also contribute to our observed findings, as in *Pastinaca sativa*, where seedlings from smaller seeds survived drought better than those from larger seeds (Hendrix, Nielsen, Nielsen, & Schutt, 1991). However, the opposite pattern has been documented within multiple species of *Glycine* in Australia (Murray et al., 2003), where seed size tends to increase along an aridity gradient. Few other studies have investigated patterns of within‐species variation in fruit or seed size along aridity gradients; thus, the expected patterns are not particularly clear. Additional complications arise from the possibility that climatic conditions may also influence seed predators, which in some systems remove more seeds where evapotranspiration and/or mean annual precipitation is greater (Orrock et al., 2015; Peco, Laffan, & Moles, 2014). For example, the likelihood of a fruit‐caching association between giant ragweed and the earthworm *L. terrestris* is higher in sites with greater fall and winter precipitation (Schutte et al., 2010), perhaps leading to regional variation in the strength of earthworm‐mediated selection pressure.

What about regional variation in fecundity? Without constraints, greater fecundity will always be favored in an annual species like giant ragweed (Smith & Fretwell, 1974; Westoby, Jurado, & Leishman, 1992); thus, the fecundity differences we see likely reflect variation in potential reproductive output given region‐specific genetic constraints on fruit size. In other words, fecundity variation is probably just a by‐product of evolved differences in fruit size across the range. Yet, even if fecundity is only an indirect target of selection, we argue that the resultant patterns could have important implications for the species' population dynamics and evolutionary potential. In particular, an increase in fecundity from east to west may effectively result in a propagule pressure gradient that increases from the core to the edge of giant ragweed's weedy range. Propagule pressure is a known driver of invasion success (Lockwood, Cassey, & Blackburn, 2005; Simberloff, 2009); thus, it stands to reason that populations in which giant ragweed produces large numbers of relatively small seeds will be well suited for dispersal and subsequent establishment of new populations. In addition, population explosions in giant ragweed's western range could interact with its high within‐population phenotypic variability, yielding rapid evolutionary responses to novel selective pressures such as exposure to pesticides and global change. In fact, current anecdotal evidence suggests that the lag between when giant ragweed first invades agricultural fields and when herbicide resistance first appears has been shorter in the west versus the east (Regnier et al., 2016). We are unaware of other range‐expanding species with spatially structured variation in propagule pressure, but we believe further study on such patterns and their consequences will yield valuable insights for basic questions in evolutionary ecology as well as weedy and invasive species management.

### 4.2 Habitat‐based variation in emergence timing, plant size and biomass allocation

Our finding that agricultural populations had delayed emergence relative to riparian populations is consistent with data from previous studies at smaller spatial scales comparing giant ragweed populations from agricultural versus successional upland or lowland sites (Illinois: Hartnett et al., 1987; Davis et al., 2013; central Ohio: Schutte et al., 2012). These habitat differences therefore appear to be pervasive and recurrent across giant ragweed's range. Rapid emergence in riparian populations probably results from strong selection for early growth in natural vegetation due to greater early season competition for light compared to cultivated fields (Hartnett et al., 1987). Evolved habitat‐based differences in life‐history timing based on competitive environment have also been identified in the weedy species *Abutilon theophrasti* (Weinig, 2005) and are consistent with recent work in *Brassica rapa* (Weis, Turner, Petro, Austen, & Wadgymar, 2015), suggesting this may be a common pattern across weedy species.

The emergence phenology of individuals from our agricultural populations could additionally reflect selection due to the timing of early spring herbicide applications in those sites. Our analyses focused only on differences in average emergence, but previous work with giant ragweed has also documented an extended emergence period and a later, secondary peak of emergence in agricultural compared to successional populations (Schutte et al., 2012). Emergence patterns from our agricultural populations were similar in both respects, including a secondary emergence peak (15–20 days after sowing) in agricultural populations only (data not shown). Although these temporal patterns may be influenced by selection imposed by herbicide applications, we do not yet have herbicide resistance data for our agricultural populations.

Larger plants tend to be more successful than smaller plants in competitive environments with dense neighboring vegetation (Gaudet & Keddy, 1988), consistent with our finding that giant ragweed from riparian populations was taller and produced more biomass compared to agricultural populations. Resources allocated to vegetative growth trade‐off with resources allocated to reproduction, so in our agricultural ragweed populations growing large may be relatively less advantageous than it is in riparian populations, allowing for enhanced reproductive allocation. Similar patterns have been observed in the perennial weed *Rumex acetosella*, which allocates more biomass to reproduction in early‐successional sites and more biomass to growth in later successional sites (Houssard & Escarré, 1995).

Surprisingly, we identified these habitat‐based differences even though gene flow between paired populations is expected to be common. Giant ragweed is wind‐pollinated and our average interpopulation distances were not great (average distance within region: 3.2 km), potentially leading to admixture between populations from contrasting habitats. Ragweed fruits can also disperse long distances, both naturally by flotation or anthropogenically along major transportation routes (Follak, Dullinger, Kleinbauer, Moser, & Essl, 2013; Payne, 1970; Regnier et al., 2016); either should contribute to population homogenization (Loveless & Hamrick, 1984). Local adaptation can occur even at fine spatial scales (Hereford, 2009; Houssard & Escarré, 1995); thus, if habitat‐specific selective pressures on emergence timing are strong enough to counter any homogenizing effects of gene flow in our system, then local adaptation may be a possible outcome. These findings highlight the utility of agroecosystems as natural laboratories for investigating questions in evolutionary ecology, particularly as they pertain to common weedy plant species (Vigueira et al., 2013).

### 4.3 Patterns of variation in fruit size and morphology

Our observation that individual fruit mass was greater in plants from agricultural versus riparian populations was surprising in light of expectations for larger seeds to be adaptive in densely vegetated habitats (Leishman et al., 2000). But competition from neighboring vegetation may have negligible effects on selection for seed size when the predominant strategy is competition avoidance via rapid seedling emergence, as appears to be the case for giant ragweed. In this system, smaller fruits may instead be favored in riparian vegetation because of strong selection from postdispersal seed predators, which often remove more seeds from heavily vegetated sites compared to agricultural habitats (Mittelbach & Gross, 1984). Smaller ragweed fruits may be more likely to escape predation by becoming buried more readily than larger fruits (Benvenuti, 2007; Chambers, MacMahon, & Haefner, 1991; Regnier et al., 2008), or they may be more resistant to predation because of scaling relationships that lead to increased specific toughness (Fricke & Wright, 2016).

At this point, we can only speculate about the underlying mechanisms driving habitat‐specific variation in fruit size; however, the hypotheses outlined above do suggest that selection on seed size would be stronger in riparian than in agricultural populations. Accordingly, heritability for seed size was also greater in plants from riparian populations compared to agricultural ones (though not significantly so), and heritability was significantly positive only for riparian populations. Of course, this distinction may alternatively result from neutral processes (e.g., larger effective population sizes in riparian habitats) or from selective sweeps in agricultural conditions that eliminated preexisting heritable variation. And, seed size variation may result from selective agents not outlined here, including local and historical tillage patterns (reviewed by Gherza & Martínez‐Gherza, 2000). Reciprocal transplant experiments designed to permit the estimation of selection differentials on seed size (and correlated traits) in giant ragweed from agricultural versus riparian populations would help to clarify the mechanisms ultimately driving these observations.

After correcting for fruit size differences, all other aspects of fruit morphology we measured were similar between habitats, suggesting that variability in surface texture may not play a major role in driving the habitat‐related differences we observed in giant ragweed. Variation in diaspore surface texture has received only limited attention in the literature; thus, it is unclear whether increased surface texture should be adaptive under certain circumstances. Possible implications of increasingly textured fruits or seeds include reduced seed burial in response to freeze–thaw action (Benvenuti, 2007; Leishman et al., 2000) and perhaps increased predation resistance due to increased handling time. In our samples, larger seeds had a greater degree of surface texture. This correlation may be entirely nonadaptive, but given that the smaller and less textured seeds that were more prevalent in our westernmost populations would be expected to become buried more readily and also that smaller seeds are expected to persist longer in the seed bank than larger seeds (Harrison, Regnier, Schmoll, & Harrison, 2007; Schutte, Regnier, & Harrison, 2008; Venable & Brown, 1988), such variation in surface texture may have played some role (albeit weak) in facilitating the spread of giant ragweed across its range.

### 4.4 Plastic phenotypes versus local adaptation in weedy plant species?

The presence of continental‐ and local‐scale variation in giant ragweed conflicts with the expectation that weedy and invasive species succeed primarily because they have a high degree of phenotypic plasticity (Davidson et al., 2011; Richards et al., 2006) or because they have “general‐purpose genotypes” (Baker, 1965). Despite empirical data supporting the occurrence of general‐purpose genotypes (Hermanutz & Weaver, 1996; Parker et al., 2003), local differentiation and putative adaptation has also been highlighted in many weedy and invasive plant taxa (Begg, Wishart, Young, Squire, & Iannetta, 2012; Clements et al., 2004; Joshi et al., 2001; Kane & Rieseberg, 2008; Maron, Vilà, Bommarco, Elmendorf, & Beardsley, 2004; Warwick et al., 1987; Weinig, 2005). Our results emphasize that even in a system where local adaptation seems unlikely—here, a primarily outcrossing species that is known to be extremely plastic morphologically (Abul‐Fatih et al., 1979)—local adaptation may occur.

### 4.5 Mosaic patterns of life‐history trait variance: Evolutionary potential in weedy and invasive species

The geographic pattern of phenotypic variation we see in giant ragweed reflects a mosaic of presumably strong and variable selection pressures across its range. Geographically structured variability in life‐history traits across a species' range is rarely documented but may be common, at least for widespread and genetically diverse species occurring across habitats with sharply contrasting environmental conditions. In plants, one trait that does show a similarly complex geographic structure is herbicide resistance (Baucom & Mauricio, 2008; Délye et al., 2010). This trait is clearly adaptive and reflects exceptionally strong selection pressures applied differentially at small scales (i.e., individual farm fields) across the landscape. Population differentiation can therefore occur even if substantial potential for gene flow exists among populations. The patterns in giant ragweed are more complex because they involve multiple traits, but we can infer that the underlying selection pressures must be similarly strong and finely structured across its range to offset likely gene flow in this wind‐pollinated and widely occurring weedy species.

Geographic patterns of intraspecific phenotypic variation may represent the consequences of strong selection, but they also set the stage for future evolutionary change. The right combination of traits may permit a species to respond quickly to novel selection pressures, thus representing a hotspot of evolutionary potential (sensu Thompson & Cunningham, 2002). As noted above, the western portion of giant ragweed's weedy range might represent such an evolutionary hotspot due to the greater reproductive output and therefore increased opportunities for rare variants to occur and establish there. We hypothesize that naturally occurring variation in key life‐history traits is an important aspect of giant ragweed's success as a broadly distributed and range‐expanding species, and we predict that other successful weedy and invasive species may show similar patterns.

## CONFLICT OF INTEREST

None declared.

## DATA ARCHIVING STATEMENT

All data from this article are available from The Knowledge Network for Biocomplexity (https://doi.org/10.5063/f1z036b7).

## References

[eva12614-bib-0001] Abul‐Fatih, H. A. , Bazzaz, F. A. , & Hunt, R. (1979). The biology of *Ambrosia trifida* L. New Phytologist, 83, 829–838. https://doi.org/10.1111/j.1469-8137.1979.tb02314.x

[eva12614-bib-0002] Ågren, J. (1989). Seed size and number in *Rubus chamaemorus*: Between‐habitat variation, and effects of defoliation and supplemental pollination. Journal of Ecology, 77, 1080–1092.

[eva12614-bib-0003] Albert, C. H. , Grassein, F. , Schurr, F. M. , Vieilledent, G. , & Violle, C. (2011). When and how should intraspecific variability be considered in trait‐based plant ecology? Perspectives in Plant Ecology, Evolution and Systematics, 13, 217–225. https://doi.org/10.1016/j.ppees.2011.04.003

[eva12614-bib-0004] Baker, H. G. (1965). Characteristics and modes of origin of weeds In Baker H., & Stebbins G. (Eds.), The genetics of colonizing species (pp. 147–168). New York, NY: Academic Press.

[eva12614-bib-0005] Barnett, K. A. , & Steckel, L. E. (2013). Giant ragweed (*Ambrosia trifida*) competition in cotton. Weed Science, 61, 543–548. https://doi.org/10.1614/WS-D-12-00169.1

[eva12614-bib-0006] Bassett, I. J. , & Crompton, C. W. (1982). The biology of Canadian weeds: *Ambrosia trifida* L. Canadian Journal of Plant Science, 62, 1003–1010. https://doi.org/10.4141/cjps82-148

[eva12614-bib-0007] Baucom, R. S. , & Mauricio, R. (2008). The evolution of novel herbicide tolerance in a noxious weed: The geographic mosaic of selection. Evolutionary Ecology, 22, 85–101. https://doi.org/10.1007/s10682-007-9160-1

[eva12614-bib-0008] Begg, G. S. , Wishart, J. , Young, M. W. , Squire, G. R. , & Iannetta, P. P. M. (2012). Genetic structure among arable populations of *Capsella bursa‐pastoris* is linked to functional traits and in‐field conditions. Ecography, 35, 446–457. https://doi.org/10.1111/j.1600-0587.2011.07030.x

[eva12614-bib-0009] Benvenuti, S. (2007). Natural weed seed burial: Effect of soil texture, rain and seed characteristics. Seed Science Research, 17, 211–219. https://doi.org/10.1017/S0960258507782752

[eva12614-bib-0010] Bhattarai, G. P. , Meyerson, L. A. , Anderson, J. , Cummings, D. , Allen, W. J. , & Cronin, J. T. (2017). Biogeography of a plant invasion: Genetic variation and plasticity in latitudinal clines for traits related to herbivory. Ecological Monographs, 87, 57–75. https://doi.org/10.1002/ecm.1233

[eva12614-bib-0011] Chambers, J. C. , MacMahon, J. A. , & Haefner, J. H. (1991). Seed entrapment in alpine ecosystems: Effects of soil particle size and diaspore morphology. Ecology, 72, 1668–1677. https://doi.org/10.2307/1940966

[eva12614-bib-0012] Clements, D. R. , DiTommaso, A. , Jordan, N. , Booth, B. D. , Cardina, J. , Doohan, D. , … Swanton, C. J. (2004). Adaptability of plants invading North American cropland. Agriculture, Ecosystems & Environment, 104, 379–398. https://doi.org/10.1016/j.agee.2004.03.003

[eva12614-bib-0013] Davidson, A. M. , Jennions, M. , & Nicotra, A. B. (2011). Do invasive species show higher phenotypic plasticity than native species and if so, is it adaptive? A meta‐analysis. Ecology Letters, 14, 419–431. https://doi.org/10.1111/j.1461-0248.2011.01596.x 21314880 10.1111/j.1461-0248.2011.01596.x

[eva12614-bib-0014] Davis, A. S. , Clay, S. , Cardina, J. , Dille, A. , Forcella, F. , Lindquist, J. , & Sprague, C. (2013). Seed burial physical environment explains departures from regional hydrothermal model of giant ragweed (*Ambrosia trifida*) seedling emergence in U.S. Midwest. Weed Science, 61, 415–421. https://doi.org/10.1614/WS-D-12-00139.1

[eva12614-bib-0015] De Jong, G. (2005). Evolution of phenotypic plasticity: Patterns of plasticity and the emergence of ecotypes. New Phytologist, 166, 101–118. https://doi.org/10.1111/j.1469-8137.2005.01322.x 15760355 10.1111/j.1469-8137.2005.01322.x

[eva12614-bib-0016] Del'Aquila, A. (2007). Towards new computer imaging techniques applied to seed quality testing and sorting. Seed Science and Technology, 35, 519–538. https://doi.org/10.15258/sst

[eva12614-bib-0017] Délye, C. , Michel, S. , Bérard, A. , Chauvel, B. , Brunel, D. , Guillemin, J.‐P. , … Le Corre, V. (2010). Geographical variation in resistance to acetyl‐coenzyme A carboxylase‐inhibiting herbicides across the range of the arable weed *Alopecurus myosuroides* (black‐grass). New Phytologist, 186, 1005–1017. https://doi.org/10.1111/j.1469-8137.2010.03233.x 20345631 10.1111/j.1469-8137.2010.03233.x

[eva12614-bib-0018] Dlugosch, K. M. , & Parker, I. M. (2008). Founding events in species invasions: Genetic variation, adaptive evolution, and the role of multiple introductions. Molecular Ecology, 17, 431–449. https://doi.org/10.1111/j.1365-294X.2007.03538.x 17908213 10.1111/j.1365-294X.2007.03538.x

[eva12614-bib-0019] Eriksson, O. (1999). Seed size variation and its effect on germination and seedling performance in the clonal herb *Convallaria majalis* . Acta Oecologica, 20, 61–66. https://doi.org/10.1016/S1146-609X(99)80016-2

[eva12614-bib-0020] Fenner, M. , & Thompson, K. (2005). The ecology of seeds. Cambridge, UK: Cambridge University Press https://doi.org/10.1017/CBO9780511614101

[eva12614-bib-0021] Follak, S. , Dullinger, S. , Kleinbauer, I. , Moser, D. , & Essl, F. (2013). Invasion dynamics of three allergenic invasive Asteraceae (*Ambrosia trifida*,* Artemisia annua*,* Iva xanthiifolia*) in central and eastern Europe. Preslia, 85, 41–61.

[eva12614-bib-0022] Forsman, A. (2014). Effects of genotypic and phenotypic variation on establishment are important for conservation, invasion, and infection biology. Proceedings of the National Academy of Sciences, 111, 302–307. https://doi.org/10.1073/pnas.1317745111 10.1073/pnas.1317745111 PMC3890895 24367109

[eva12614-bib-0023] Fricke, E. C. , & Wright, S. J. (2016). The mechanical defence advantage of small seeds. Ecology Letters, 19, 987–991. https://doi.org/10.1111/ele.12637 27324185 10.1111/ele.12637

[eva12614-bib-0024] Ganie, Z. A. , Lindquist, J. L. , Jugulam, M. , Kruger, G. R. , Marx, D. B. , & Jhala, A. J. (2017). An integrated approach to control glyphosate‐resistant *Ambrosia trifida* with tillage and herbicides in glyphosate‐resistant maize. Weed Research, 57, 112–122. https://doi.org/10.1111/wre.12244

[eva12614-bib-0025] Gaudet, C. L. , & Keddy, P. A. (1988). A comparative approach to predicting competitive ability from plant traits. Nature, 334, 242–243. https://doi.org/10.1038/334242a0

[eva12614-bib-0026] Gherza, C. M. , & Martínez‐Gherza, M. A. (2000). Ecological correlates of weed seed size and persistence in the soil under different tilling systems: Implications for weed management. Field Crops Research, 67, 141–148. https://doi.org/10.1016/S0378-4290(00)00089-7

[eva12614-bib-0027] Gitz, D. C. , Baker, J. T. , Xin, Z. , Burke, J. J. , & Lascano, R. J. (2015). The microenvironment within and pollen transmission through polyethylene sorghum pollination bags. American Journal of Plant Sciences, 06, 265–274. https://doi.org/10.4236/ajps.2015.62030

[eva12614-bib-0028] Harper, J. L. , Lovell, P. H. , & Moore, K. G. (1970). The shapes and sizes of seeds. Annual Review of Ecology and Systematics, 1, 327–356. https://doi.org/10.1146/annurev.es.01.110170.001551

[eva12614-bib-0029] Harrison, S. K. , Regnier, E. E. , & Schmoll, J. T. (2003). Postdispersal predation of giant ragweed (*Ambrosia trifida*) seed in no‐tillage corn. Weed Science, 51, 955–964. https://doi.org/10.1614/P2002-110

[eva12614-bib-0030] Harrison, S. K. , Regnier, E. E. , Schmoll, J. T. , & Harrison, J. M. (2007). Seed size and burial effects on giant ragweed (*Ambrosia trifida*) emergence and seed demise. Weed Science, 55, 16–22. https://doi.org/10.1614/WS-06-109.1

[eva12614-bib-0031] Harrison, S. K. , Regnier, E. E. , Schmoll, J. T. , & Webb, J. E. (2001). Competition and fecundity of giant ragweed in corn. Weed Science, 49, 224–229. https://doi.org/10.1614/0043-1745(2001)049[0224:CAFOGR]2.0.CO;2

[eva12614-bib-0032] Hartnett, D. C. , Hartnett, B. B. , & Bazzaz, F. A. (1987). Persistence of *Ambrosia trifida* populations in old fields and responses to successional changes. American Journal of Botany, 74, 1239–1248. https://doi.org/10.1002/j.1537-2197.1987.tb08737.x

[eva12614-bib-0033] Heap, I. (2018). The international survey of herbicide resistant weeds. Retrieved from http://www.weedscience.org. Online. Accessed January 18, 2018.

[eva12614-bib-0034] Hendrix, S. D. , Nielsen, E. , Nielsen, T. , & Schutt, M. (1991). Are seedlings from small seeds always inferior to seedlings from large seeds? Effects of seed biomass on seedling growth in *Pastinaca sativa* L. New Phytologist, 119, 299–305. https://doi.org/10.1111/j.1469-8137.1991.tb01034.x 10.1111/j.1469-8137.1991.tb01034.x 33874142

[eva12614-bib-0035] Hereford, J. (2009). A quantitative survey of local adaptation and fitness trade‐offs. The American Naturalist, 173, 579–588. https://doi.org/10.1086/597611 10.1086/597611 19272016

[eva12614-bib-0036] Hermanutz, L. A. , & Weaver, S. E. (1996). Agroecotypes or phenotypic plasticity? Comparison of agrestal and ruderal populations of the weed *Solanum ptycanthum* . Oecologia, 105, 271–280. https://doi.org/10.1007/BF00328557 28307093 10.1007/BF00328557

[eva12614-bib-0037] Houssard, C. , & Escarré, J. (1995). Variation and covariation among life‐history traits in *Rumex acetosella* from a successional old‐field gradient. Oecologia, 102, 70–80. https://doi.org/10.1007/BF00333312 28306809 10.1007/BF00333312

[eva12614-bib-0038] Jakobsson, A. , & Eriksson, O. (2000). A comparative study of seed number, seed size, seedling size and recruitment in grassland plants. Oikos, 88, 494–502. https://doi.org/10.1034/j.1600-0706.2000.880304.x

[eva12614-bib-0039] Joshi, J. , Schmid, B. , Caldeira, M. C. , Dimitrakopoulos, P. G. , Good, J. , Harris, R. , … Lawton, J. H. (2001). Local adaptation enhances performance of common plant species. Ecology Letters, 4, 536–544. https://doi.org/10.1046/j.1461-0248.2001.00262.x

[eva12614-bib-0040] Kane, N. C. , & Rieseberg, L. H. (2008). Genetics and evolution of weedy *Helianthus annuus* populations: Adaptation of an agricultural weed. Molecular Ecology, 17, 384–394. https://doi.org/10.1111/j.1365-294X.2007.03467.x 17725567 10.1111/j.1365-294X.2007.03467.x

[eva12614-bib-0041] Kittelson, P. M. , & Maron, J. L. (2001). Fine‐scale genetically based differentiation of life‐history traits in the perennial shrub *Lupinus arboreus* . Evolution, 55, 2429–2438. https://doi.org/10.1111/j.0014-3820.2001.tb00758.x 11831659 10.1111/j.0014-3820.2001.tb00758.x

[eva12614-bib-0042] Lavergne, S. , & Molofsky, J. (2007). Increased genetic variation and evolutionary potential drive the success of an invasive grass. Proceedings of the National Academy of Sciences of the United States of America, 104, 3883–3888. https://doi.org/10.1073/pnas.0607324104 17360447 10.1073/pnas.0607324104 PMC1805698

[eva12614-bib-0043] Lázaro, A. , & Traveset, A. (2009). Does the spatial variation in selective pressures explain among‐site differences in seed mass? A test with *Buxus balearica* . Evolutionary Ecology, 23, 847 https://doi.org/10.1007/s10682-008-9275-z

[eva12614-bib-0044] Lee, C. E. , & Gelembiuk, G. W. (2008). Evolutionary origins of invasive populations. Evolutionary Applications, 1, 427–448. https://doi.org/10.1111/j.1752-4571.2008.00039.x 25567726 10.1111/j.1752-4571.2008.00039.x PMC3352381

[eva12614-bib-0045] Leishman, M. , Wright, I. , Moles, A. , & Westoby, M. (2000). The evolutionary ecology of seed size In Fenner M. (Ed.), Seeds: The ecology of regeneration in plant communities (pp. 31–57). 2nd ed New York, NY: CAB International https://doi.org/10.1079/9780851994321.0000

[eva12614-bib-0046] Lockwood, J. L. , Cassey, P. , & Blackburn, T. (2005). The role of propagule pressure in explaining species invasions. Trends in Ecology & Evolution, 20, 223–228. https://doi.org/10.1016/j.tree.2005.02.004 16701373 10.1016/j.tree.2005.02.004

[eva12614-bib-0047] Loveless, M. D. , & Hamrick, J. L. (1984). Ecological determinants of genetic structure in plant populations. Annual Review of Ecology and Systematics, 15, 65–95. https://doi.org/10.1146/annurev.es.15.110184.000433

[eva12614-bib-0048] Maron, J. L. , Vilà, M. , Bommarco, R. , Elmendorf, S. , & Beardsley, P. (2004). Rapid evolution of an invasive plant. Ecological Monographs, 74, 261–280. https://doi.org/10.1890/03-4027

[eva12614-bib-0049] Michaels, H. J. , Benner, B. , Hartgerink, A. P. , Lee, T. D. , Rice, S. , Willson, M. F. , & Bertin, R. I. (1988). Seed size variation: Magnitude, distribution, and ecological correlates. Evolutionary Ecology, 2, 157–166. https://doi.org/10.1007/BF02067274

[eva12614-bib-0050] Mittelbach, G. G. , & Gross, K. L. (1984). Experimental studies of seed predation in old‐fields. Oecologia, 65, 7–13. https://doi.org/10.1007/BF00384455 28312102 10.1007/BF00384455

[eva12614-bib-0051] Mojonnier, L. (1998). Natural selection on two seed‐size traits in the common morning glory Ipomoea purpurea (Convolvulaceae): Patterns and evolutionary consequences. The American Naturalist, 152, 188–203. https://doi.org/10.1086/286161 10.1086/286161 18811385

[eva12614-bib-0052] Moles, A. T. , Falster, D. S. , Leishman, M. R. , & Westoby, M. (2004). Small‐seeded species produce more seeds per square metre of canopy per year, but not per individual per lifetime. Journal of Ecology, 92, 384–396. https://doi.org/10.1111/j.0022-0477.2004.00880.x

[eva12614-bib-0053] Murray, B. R. , Brown, A. H. D. , & Grace, J. P. (2003). Geographic gradients in seed size among and within perennial Australian *Glycine* species. Australian Journal of Botany, 51, 47 https://doi.org/10.1071/BT02069

[eva12614-bib-0054] Nelson, M. F. , & Anderson, N. O. (2015). Variation among genotypes and source habitats in growth and fecundity of the wetland invasive plant *Phalaris arundinacea* L. Wetlands, 35, 1175–1184. https://doi.org/10.1007/s13157-015-0704-9

[eva12614-bib-0055] Olson, E. (2011). Particle shape factors and their use in image analysis‐part 1: Theory. Journal of GXP Compliance, 15, 85–96.

[eva12614-bib-0056] Orrock, J. L. , Borer, E. T. , Brudvig, L. A. , Firn, J. , MacDougall, A. S. , Melbourne, B. A. , … Seabloom, E. W. (2015). A continent‐wide study reveals clear relationships between regional abiotic conditions and post‐dispersal seed predation. Journal of Biogeography, 42, 662–670. https://doi.org/10.1111/jbi.12451

[eva12614-bib-0057] Parciak, W. (2002). Environmental variation in seed number, size, and dispersal of a fleshy‐fruited plant. Ecology, 83, 780–793. https://doi.org/10.1890/0012-9658(2002)083[0780:EVISNS]2.0.CO;2

[eva12614-bib-0058] Parker, I. M. , Rodriguez, J. , & Loik, M. E. (2003). An evolutionary approach to understanding the biology of invasions: Local adaptation and general‐purpose genotypes in the weed *Verbascum thapsus* . Conservation Biology, 17, 59–72. https://doi.org/10.1046/j.1523-1739.2003.02019.x

[eva12614-bib-0059] Patzoldt, W. L. , & Tranel, P. J. (2002). Molecular analysis of cloransulam resistance in a population of giant ragweed. Weed Science, 50, 299–305. https://doi.org/10.1614/0043-1745(2002)050[0299:MAOCRI]2.0.CO;2

[eva12614-bib-0060] Paul‐Victor, C. , & Turnbull, L. A. (2009). The effect of growth conditions on the seed size/number trade‐off. PLoS ONE, 4, 1–10. https://doi.org/10.1371/journal.pone.0006917 10.1371/journal.pone.0006917 PMC2735032 19746162

[eva12614-bib-0061] Payne, W. W. (1970). Preliminary reports on the flora of Wisconsin No. 62—Compositae VI. Composite family VI. The genus *Ambrosia*—the ragweeds. Transactions of the Wisconsin Academy of Sciences, Arts and Letters, 58, 353–371.

[eva12614-bib-0062] Payne, W. W. , & Jones, V. H. (1962). The taxonomic status and archeological significance of a giant ragweed from prehistoric bluff shelters in the Ozark Plateau region. Papers of the Michigan Academy of Science, Arts, and Letters, 47, 147–163.

[eva12614-bib-0063] Peco, B. , Laffan, S. W. , & Moles, A. T. (2014). Global patterns in post‐dispersal seed removal by invertebrates and vertebrates. PLoS ONE, 9, e91256 https://doi.org/10.1371/journal.pone.0091256 24618879 10.1371/journal.pone.0091256 PMC3949765

[eva12614-bib-0064] Pyšek, P. , & Richardson, D. M. (2008). Traits associated with invasiveness in alien plants: Where do we stand? In Nentwig D. W. (Ed.), Biological invasions (pp. 97–125). Berlin Heidelberg: Springer.

[eva12614-bib-0065] R Core Team (2016). R: A language and environment for statistical computing. Vienna, Austria: R Foundation for Statistical Computing.

[eva12614-bib-0066] Rasband, W. S. (1997). ImageJ. Bethesda, Maryland, USA: U. S. National Institutes of Health.

[eva12614-bib-0067] Regnier, E. , Harrison, S. K. , Liu, J. , Schmoll, J. T. , Edwards, C. A. , Arancon, N. , & Holloman, C. (2008). Impact of an exotic earthworm on seed dispersal of an indigenous US weed. Journal of Applied Ecology, 45, 1621–1629. https://doi.org/10.1111/j.1365-2664.2008.01489.x

[eva12614-bib-0068] Regnier, E. E. , Harrison, S. K. , Loux, M. M. , Holloman, C. , Venkatesh, R. , Diekmann, F. , … Johnson, W. G. (2016). Certified crop advisors' perceptions of giant ragweed (*Ambrosia trifida*) distribution, herbicide resistance, and management in the Corn Belt. Weed Science, 64, 361–377. https://doi.org/10.1614/WS-D-15-00116.1

[eva12614-bib-0069] Richards, C. L. , Bossdorf, O. , Muth, N. Z. , Gurevitch, J. , & Pigliucci, M. (2006). Jack of all trades, master of some? On the role of phenotypic plasticity in plant invasions. Ecology Letters, 9, 981–993. https://doi.org/10.1111/j.1461-0248.2006.00950.x 16913942 10.1111/j.1461-0248.2006.00950.x

[eva12614-bib-0070] Sako, Y. , Regnier, E. E. , Daoust, T. , Fujimura, K. , Kent Harrison, S. , & McDonald, M. B. (2001). Computer image analysis and classification of giant ragweed seeds. Weed Science, 49, 738–745. https://doi.org/10.1614/0043-1745(2001)049[0738:CIAACO]2.0.CO;2

[eva12614-bib-0071] SAS Institute (2008). SAS. Cary, NC: SAS Institute.

[eva12614-bib-0072] Schutte, B. J. , Liu, J. , Davis, A. S. , Harrison, S. K. , & Regnier, E. E. (2010). Environmental factors that influence the association of an earthworm (*Lumbricus terrestris* L.) and an annual weed (*Ambrosia trifida* L.) in no‐till agricultural fields across the eastern U.S. Corn Belt. Agriculture, Ecosystems & Environment, 138, 197–205. https://doi.org/10.1016/j.agee.2010.05.001

[eva12614-bib-0073] Schutte, B. J. , Regnier, E. E. , & Harrison, S. K. (2008). The association between seed size and seed longevity among maternal families in *Ambrosia trifida* L. populations. Seed Science Research, 18, 201–211. https://doi.org/10.1017/S0960258508082974

[eva12614-bib-0074] Schutte, B. J. , Regnier, E. E. , & Harrison, S. K. (2012). Seed dormancy and adaptive seedling emergence timing in giant ragweed (*Ambrosia trifida*). Weed Science, 60, 19–26. https://doi.org/10.1614/WS-D-11-00049.1

[eva12614-bib-0075] Simberloff, D. (2009). The role of propagule pressure in biological invasions. Annual Review of Ecology, Evolution, and Systematics, 40, 81–102. https://doi.org/10.1146/annurev.ecolsys.110308.120304

[eva12614-bib-0076] Smith, C. C. , & Fretwell, S. D. (1974). The optimal balance between size and number of offspring. The American Naturalist, 108, 499–506. https://doi.org/10.1086/282929

[eva12614-bib-0077] Smith, D. C. , & Mehienbacher, S. A. (1994). Use of Tyvek housewrap for pollination bags in breeding of hazelnut. HortScience, 29, 506–506.

[eva12614-bib-0078] Sõber, V. , & Ramula, S. (2013). Seed number and environmental conditions do not explain seed size variability for the invasive herb *Lupinus polyphyllus* . Plant Ecology, 214, 883–892. https://doi.org/10.1007/s11258-013-0216-8

[eva12614-bib-0079] Thompson, J. N. , & Cunningham, B. M. (2002). Geographic structure and dynamics of coevolutionary selection. Nature, 417, 735–738. https://doi.org/10.1038/nature00810 12066183 10.1038/nature00810

[eva12614-bib-0080] Turnbull, L. A. , Rees, M. , & Crawley, M. J. (1999). Seed mass and the competition/colonization trade‐off: A sowing experiment. Journal of Ecology, 87, 899–912. https://doi.org/10.1046/j.1365-2745.1999.00405.x

[eva12614-bib-0081] Van Kleunen, M. , Weber, E. , & Fischer, M. (2010). A meta‐analysis of trait differences between invasive and non‐invasive plant species. Ecology Letters, 13, 235–245. https://doi.org/10.1111/j.1461-0248.2009.01418.x 20002494 10.1111/j.1461-0248.2009.01418.x

[eva12614-bib-0082] Venable, D. L. , & Brown, J. S. (1988). The selective interactions of dispersal, dormancy, and seed size as adaptations for reducing risk in variable environments. The American Naturalist, 131, 360–384. https://doi.org/10.1086/284795

[eva12614-bib-0083] Vigueira, C. C. , Olsen, K. M. , & Caicedo, A. L. (2013). The red queen in the corn: Agricultural weeds as models of rapid adaptive evolution. Heredity, 110, 303–311. https://doi.org/10.1038/hdy.2012.104 23188175 10.1038/hdy.2012.104 PMC3607111

[eva12614-bib-0084] Warwick, S. I. , Thompson, B. K. , & Black, L. D. (1987). Genetic variation in Canadian and European populations of the colonizing weed species *Apera spica‐venti* . New Phytologist, 106, 301–317. https://doi.org/10.1111/j.1469-8137.1987.tb00145.x

[eva12614-bib-0085] Webster, T. M. , Loux, M. M. , Regnier, E. E. , & Harrison, S. K. (1994). Giant ragweed (*Ambrosia trifida*) canopy architecture and interference studies in soybean (*Glycine max*). Weed Technology, 8, 559–564. http://www.jstor.org/stable/3988029

[eva12614-bib-0086] Weinig, C. (2005). Rapid evolutionary responses to selection in heterogeneous environments among agricultural and nonagricultural weeds. International Journal of Plant Sciences, 166, 641–647. https://doi.org/10.1086/429853

[eva12614-bib-0087] Weis, A. E. , Turner, K. M. , Petro, B. , Austen, E. J. , & Wadgymar, S. M. (2015). Hard and soft selection on phenology through seasonal shifts in the general and social environments: A study on plant emergence time. Evolution, 69, 1361–1374. https://doi.org/10.1111/evo.12677 25929822 10.1111/evo.12677

[eva12614-bib-0088] Westoby, M. , Jurado, E. , & Leishman, M. (1992). Comparative evolutionary ecology of seed size. Trends in Ecology & Evolution, 7, 368–372. https://doi.org/10.1016/0169-5347(92)90006-W 21236070 10.1016/0169-5347(92)90006-W

[eva12614-bib-0089] Whitney, K. D. , & Gabler, C. A. (2008). Rapid evolution in introduced species, “invasive traits” and recipient communities: Challenges for predicting invasive potential. Diversity and Distributions, 14, 569–580. https://doi.org/10.1111/j.1472-4642.2008.00473.x

[eva12614-bib-0090] Williams, J. L. , Kendall, B. E. , & Levine, J. M. (2016). Rapid evolution accelerates plant population spread in fragmented experimental landscapes. Science, 353, 482–485. https://doi.org/10.1126/science.aaf6268 27471303 10.1126/science.aaf6268

[eva12614-bib-0091] Willis, S. G. , & Hulme, P. E. (2004). Environmental severity and variation in the reproductive traits of *Impatiens glandulifera* . Functional Ecology, 18, 887–898. https://doi.org/10.1111/j.0269-8463.2004.00907.x

