# Supplementary material for: A mosaic of phenotypic variation in giant ragweed (Ambrosia trifida): Local‐ and continental‐scale patterns in a range‐expanding agricultural weed
Source: Evol Appl. 2018 Mar 14;11(6):995–1009. doi: 10.1111/eva.12614 (PMC5999201; doi:10.1111/eva.12614)

**Supporting information for: A mosaic of phenotypic variation in giant ragweed (*Ambrosia trifida*): local and continental scale patterns in a range-expanding agricultural weed**

Table S1: Correlations among metrics of fruit morphology. Data from field-collected and greenhouse-grown fruits are above and below the diagonal, respectively.

|  | Fruit Area | Fruit Mass | Solidity | Convexity | Length:Width |
| --- | --- | --- | --- | --- | --- |
| Fruit Area | --- | 0.725*** | -0.236*** | -0.336*** | -0.254*** |
| Fruit Mass | 0.753*** | --- | -0.03 | -0.123* | -0.279*** |
| Solidity | -0.116 | -0.052 | --- | 0.793*** | 0.156** |
| Convexity | -0.321*** | -0.233** | 0.676*** | --- | 0.077 |
| Length:Width | -0.160* | -0.124^ | 0.288*** | 0.140^ | --- |

p<0.001: ***, p<0.01:**, p<0.05:*, p<0.1:^

Table S2: Principal component (PC) loadings for the first three axes of PCA on fruit morphology metrics. The first three principal components accounted for 90.8% of total variation (with 43.1, 29.8 and 17.9% of total variation explained by those axes, respectively).

|  | PC1 | PC2 | PC3 |
| --- | --- | --- | --- |
| Fruit mass | 0.410 | 0.587 | 0.091 |
| Fruit area | 0.510 | 0.443 | 0.160 |
| Convexity | -0.512 | 0.411 | -0.294 |
| Solidity | -0.460 | 0.538 | -0.051 |
| Length:Width ratio | -0.313 | 0.025 | 0.937 |

Figure S1: Trait variation in greenhouse-grown giant ragweed, illustrating population means (compare with Fig 3, which shows habitat means). Populations are ordered west to east within each habitat type. Habitat-based differences are statistically significant (see Table 1). Error bars are ±1 SEM.

**
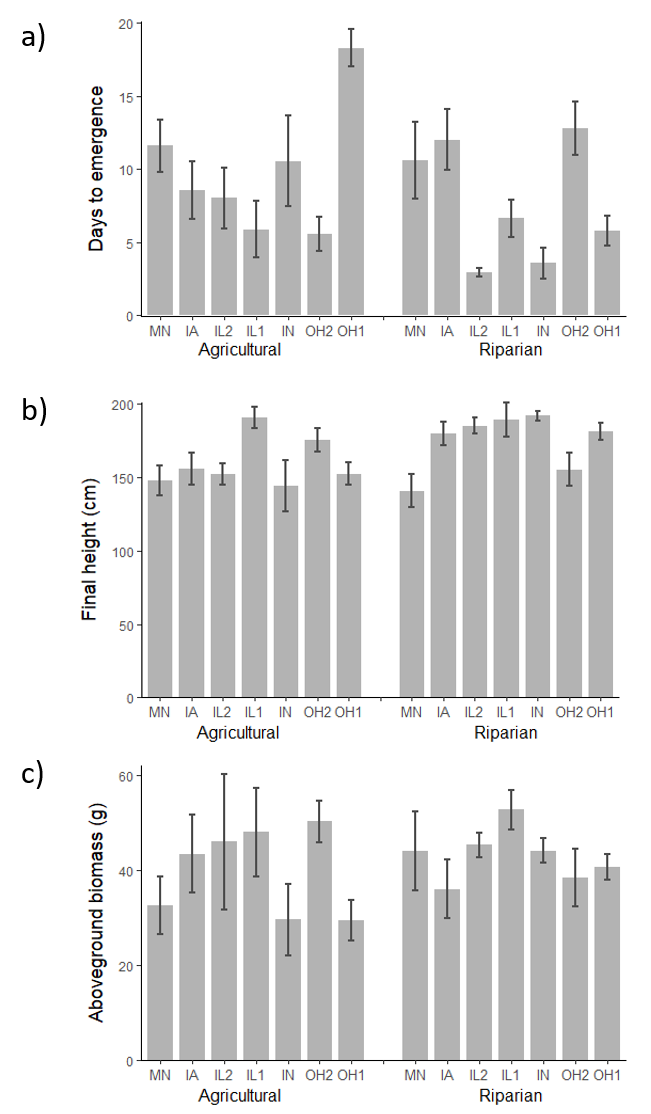
**

Figure S2: Convexity increased from east to west across our sample range on average, reflecting less fruit surface texture in the west than in the east. Data are averaged across habitat and year within each region. Error bars are ±1 SEM. The best-fit line represents model-based parameter estimates as reported in Table 2.


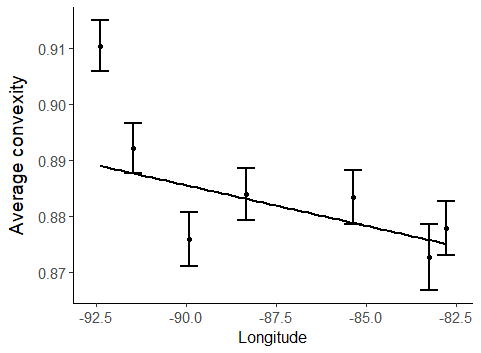


Figure S3: Field-collected fruits were more elongated than fruits from the subsequent, greenhouse-grown generation (see also Fig. 8). Data are averaged across source populations. Error bars are ±1 SEM.


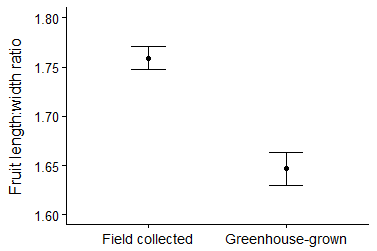

Supplement: Supplementary file 1 [file EVA-11-995-s001.docx]
